# Costs of vitamin D testing and prescribing among children in primary care

**DOI:** 10.1007/s00431-017-2986-9

**Published:** 2017-08-12

**Authors:** Emre Basatemur, Rachael Hunter, Laura Horsfall, Alastair Sutcliffe, Greta Rait

**Affiliations:** 1 Population, Policy and Practice Programme, UCL Institute of Child Health, 30 Guilford Street, London, WC1N 1EH UK; 2 Research Department of Primary Care and Population Health, University College London (Royal Free Campus), London, UK

**Keywords:** Vitamin D, Children, Healthcare costs, The Health Improvement Network, Primary care

## Abstract

Vitamin D has attracted considerable interest in recent years, with a marked increase in diagnosis of vitamin D deficiency seen among children in clinical practice in the UK. The economic implications of this change in diagnostic behaviour have not been explored. We performed a cohort study to examine longitudinal trends in healthcare expenditure arising from vitamin D testing and prescribing for children in primary care in England, using the electronic healthcare records of 722,525 children aged 0–17 years held in The Health Improvement Network database. Combined costs of vitamin D tests and prescriptions increased from £1647 per 100,000 person-years in 2008 (95% CI, £934 to £3007) to £28,913 per 100,000 person-years in 2014 (95% CI, £26,361 to £31,739). The total cost of vitamin D prescriptions and tests for children in primary care at the national level in England in 2014 was estimated to be £4.31 million (95% CI, £2.96–£6.48 million).

*Conclusion*: There has been a marked increase in healthcare expenditure on vitamin D tests and prescriptions for children in primary care over the past decade. Future research should explore the drivers for this change in diagnostic behaviour and the reasons prompting investigation of vitamin D status in clinical practice.

**Table:** 

**What is Known:** • *Vitamin D deficiency has attracted considerable interest in recent years, with a marked increase in diagnosis seen in children.* • *The economic implications of this change in diagnostic behaviour have not been explored.*
**What is New:** • *There has been a large increase in healthcare expenditure on vitamin D tests and prescriptions for children in primary care in England over the past decade (> 15 fold between 2008 and 2013).* • *Screening of vitamin D status in children without specific risk factors or clinical features of deficiency may represent avoidable healthcare expenditure.*

**Electronic supplementary material:**

The online version of this article (doi:10.1007/s00431-017-2986-9) contains supplementary material, which is available to authorized users.

## Introduction

Vitamin D has attracted considerable interest in recent years. Alongside concerns regarding a rise in cases of rickets among children in developed countries [1], a large body of observational research has stimulated debate regarding the postulated role of vitamin D deficiency (VDD) in numerous other diseases (beyond the hormone’s established functions in bone metabolism and calcium homeostasis) [15].

As vitamin D has attracted increasing attention, large increases in testing have been reported in adult practice in Australia and Canada [3]. Given the controversy regarding threshold 25-hydroxyvitamin D (25-OH-D) values used to define deficiency [7, 9, 14, 15], the high prevalence of low 25-OH-D levels in the general population, and uncertainty regarding whether treatment of biochemical VDD in asymptomatic individuals improves health outcomes, some authors have suggested that the large growth in testing in recent years reflects potential over-diagnosis and unnecessary health care costs [3, 15]. In UK children, there has been a marked increase in diagnosis of VDD in clinical practice over the last decade [2]. The aim of this study was to explore the economic implications of this change in diagnostic behaviour. Using a large population-based cohort of children in England, we examined longitudinal trends in healthcare expenditure arising from vitamin D testing and prescribing in primary care.

## Methods

A cohort study was performed using The Health Improvement Network (THIN) database, which contains anonymised electronic health records of patients registered with 639 participating UK general practices (www.epic-uk.org). A subset of THIN practices in England (*n* = 156) linked to patient-level Hospital Episode Statistics (HES) data (http://content.digital.nhs.uk/hes) were included, to maximise information regarding ethnicity.

Children aged 0–17 years, actively registered with a participating practice at any point between 2000 and 2014, were included. Children with chronic renal disease, liver disease, or conditions associated with gastrointestinal malabsorption were excluded (*n* = 3918, 0.5% of the cohort). Individuals were followed up from the latest of their date of practice registration, the date the practice met two pre-defined quality indicators for electronic data recording (acceptable mortality recording and acceptable computer usage) [10], or 1st January 2000. Exit from follow-up was the earliest of the date of transfer out of practice, the date the practice stopped contributing data to THIN, the mid-point of the 18th year after birth, date of death, or 31st December 2014.

Ethnicity was grouped into the 2001 UK Census 5-category classification, and was assigned from THIN where available, and supplemented with HES data [11]. For individuals with multiple ethnicity categories recorded (0.3% of the cohort), the most frequently recorded category was used. For children with missing ethnicity, maternal ethnicity was taken as a proxy measure, using methods described elsewhere [2].

The primary outcome was the total cost of 25-OH-D tests and prescriptions for pure preparations of calciferol (colecalciferol or ergocalciferol) in the study cohort. Prescriptions for multivitamin preparations and activated forms of vitamin D (calitriol and alfacalcidol) were excluded, as they are not indicated in the treatment of primary VDD. The unit cost of a 25-OH-D test was taken as £15, based on data from several NHS trusts. Unit costs of different calciferol preparations were derived from Prescription Cost Analysis (PCA) for England 2014, using the net ingredient cost per quantity of drug (*NIC_Qty*) [8]. Where there were multiple drugs with identical formulation and strength listed in the PCA, a weighted mean unit price was calculated using the relative frequency (*N*) with which each drug (*i*) was dispensed:

$$ \mathrm{Weighted}\  \mathrm{unit}\  \mathrm{price}=\kern0.5em \frac{\sum_i\left( NIC\_{Qty}_i\kern0.5em \times \kern0.5em {N}_i\right)}{\sum_i{N}_i} $$

Prescription costs were calculated by multiplying the weighted unit price by the quantity prescribed. Mean prescription costs were calculated for each year between 2000 and 2014, using bootstrapping to derive confidence intervals due to skewed data. Total costs of prescriptions and tests in each year were calculated by multiplying mean unit costs by rates of prescription and testing in the cohort. Multivariable Poisson regression models examined differences in rates of prescription and testing by ethnicity, age group, sex, urban or rural area of residence, and season, with inclusion of the general practice as a random effect to account for data clustering.

National costs for 2014 were estimated using the direct standardisation method. Observed costs in the cohort (per 100,000 person-years) in 2014 were stratified by age, sex, and ethnicity and applied to mid-2014 population estimates for England (www.ons.gov.uk). Analyses were performed using Stata 14.2 (StataCorp LP).

## Results

The study cohort consisted of 722,525 children from 156 practices in England, with a median follow-up time of 3.9 years (interquartile range 1.5–8.1). Descriptive characteristics are shown in Table 1.

**Table 1:** Descriptive characteristics of the study cohort (*N* = 722,525)

Characteristic	Value
Age at entry to follow-up in years, *median (IQR)*	3.9 (0.15 to 10.5)
Sex, *n* (%)	
Male	371,835 (51.5)
Female	350,690 (48.5)
Ethnicity, *n* (%)^a^	
White	499,132 (69.1)
Asian or Asian British	35,322 (4.9)
Black or Black British	25,315 (3.5)
Mixed	15,886 (2.2)
Chinese or other ethnic group	13,712 (1.9)
*Missing*	*133,158 (18.4)*
Area of residence, *n* (%)	
Urban	568,232 (78.7)
Rural	123,134 (17.0)
*Missing*	*31,159 (4.3)*

*IQR* interquartile range

^a^Ethnicity data was available from the child’s THIN or Hospital Episode Statistics record for 67.6% of the cohort, and maternal ethnicity was available as a proxy measure for 14.0%

**Fig. 1:**
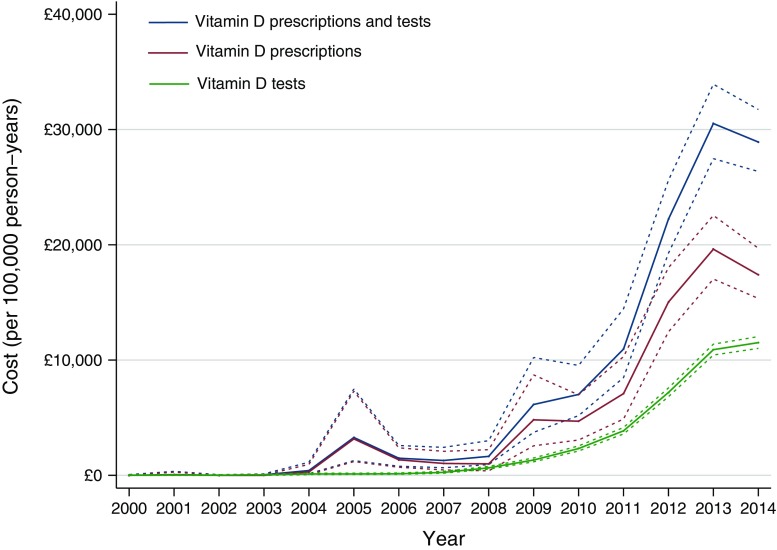
Time trends in costs arising from vitamin D prescriptions and tests in children in primary care, 2000 to 2014. (Costs of vitamin D prescriptions and tests are shown separately (*red and green lines*) as well as combined (*blue line*), *dashed lines* indicate 95% confidence intervals)

There was a marked increase in healthcare costs arising from both vitamin D tests and prescriptions in primary care after 2008 (Fig. 1 and Supplementary Table 1). Combined costs of calciferol prescriptions and 25-OH-D tests increased from £1647 per 100,000 person-years in 2008 (95% CI, £934–£3007) to £28,913 per 100,000 person-years in 2014 (95% CI, £26,361–£31,739). Rates of vitamin D testing and prescription were higher in older children, girls, and children from ethnic minority backgrounds (Supplementary Table 2). Rates of testing and prescription were marginally higher in spring compared to other seasons, and did not differ significantly by urban or rural area of residence.

Liquid formulations of calciferol accounted for 35% of prescriptions, and 76% of prescription costs. The majority of calciferol prescriptions (70%, accounting for 75% of prescription costs) were for doses higher than those recommended for prophylactic supplementation or maintenance therapy (> 1000 units/day).

Applying stratified cost rates (by age, sex, and ethnicity) to national population estimates, the total cost of vitamin D prescriptions and tests for children in primary care in England in 2014 was estimated to be £4.31 million (95% CI, £2.96–£6.48 million), of which the cost of calciferol prescriptions was £2.62 million (95% CI, £1.65–£4.24 million) and the cost of 25-OH-D tests was £1.69 million (95% CI, £1.31–£2.24 million).

## Discussion

Using a large representative sample of English children, we observed a marked increase (> 15-fold) in healthcare expenditure on vitamin D tests and prescriptions in primary care between 2008 and 2013. This mirrors trends seen in adult practice; expenditure on 25-OH-D tests in Australia is reported to have increased from 1 to 95.6 million Australian dollars between 2000 and 2010, and from 38 to 150 million Canadian dollars between 2009 and 2012 in the Ontario province of Canada [3].

THIN data is prospectively collected, and representative of real-life clinical practice. The cohort has been shown to be broadly representative of the UK general population [4]. Prescriptions and test results are particularly well recorded in electronic health data, due to computerised prescribing and electronic linkage with laboratory services. However, the study is limited to primary care and does not capture costs of testing and prescribing in secondary care. Furthermore, reliable resource use data was not available for consultations or hospital admissions, as it was not known whether episodes were primarily related to VDD or other clinical issues. Thus, although the results do not represent total healthcare costs associated with VDD diagnosis and treatment in children, they do indicate a clear trend for increasing costs over the last decade. It was not possible to investigate whether vitamin D testing and prescribing was associated with children’s body mass index, as this variable is not well recorded in THIN.

Biochemical VDD has a high prevalence in the general population, and testing is likely to identify a significant proportion of abnormal results in any patient group [15]. However, the choice of threshold 25-OH-D levels used to define deficiency varies across guidelines internationally and has a limited evidence base in children [7, 14, 15]. Whilst the benefits of treatment with pharmacological doses of vitamin D are clear in children with symptomatic deficiency, there is no evidence that testing and treating asymptomatic individuals results in improved health outcomes compared to prophylaxis with low-dose supplements [15]. For these reasons, the UK Institute for Health and Care Excellence (NICE) [13], US Endocrine Society [9], European Society for Paediatric Endocrinology [12], and European Academy of Paediatrics [7] all advise against the routine screening of vitamin D status. However, they recommend a strategy of primary prevention of deficiency through universal low-dose vitamin D supplementation for all young children and pregnant women. It has been suggested that the primary reasons for requesting 25-OH-D measurement in children should relate to symptoms of rickets/osteomalacia or muscle weakness, biochemical or radiological evidence of metabolic bone disease, hypocalcaemia, or the presence of disorders that interfere with vitamin D metabolism or absorption [15]. Testing outside of this context requires careful consideration of whether VDD is related to the child’s presentation or is a coincidental finding. Evidence that vitamin D has a clinically relevant role in the aetiology of non-musculoskeletal health outcomes is limited [7, 15].

The data available did not permit exploration of how much the increase in testing and treatment in children is being driven by improved recognition of symptomatic VDD, or by testing in other clinical situations (for example, screening of asymptomatic children, or testing prompted by the presence of non-musculoskeletal diseases that have been linked to VDD such as diabetes, atopic disorders, and infectious diseases). However, adult data suggests that the majority of vitamin D tests are performed in individuals without specific clinical features or risk factors for deficiency; the introduction of a defined set of clinical criteria permitting 25-OH-D testing in Alberta, Canada, in 2015 resulted in a 92% reduction in the number of tests ordered, and annual cost savings of almost 4 million US dollars [6]. A large reduction in healthcare expenditure on vitamin D tests has also been observed in Australia following the introduction of a policy for targeted testing [5].

In summary, there has been a large increase in healthcare expenditure on vitamin D tests and prescriptions for children in primary care over the past decade. Screening of vitamin D levels in children without specific risk factors or clinical features of deficiency may represent avoidable healthcare expenditure, and resources may be better used if directed towards improving the uptake of inexpensive multivitamin supplements by population groups at high-risk of deficiency. Future studies should explore the reasons for investigation of vitamin D status in children in clinical practice.

## Electronic supplementary material

Supplementary Table 1 (PDF 68 kb)

Supplementary Table 2 (PDF 73 kb)

## Abbreviations

25-OH-D: 25-hydroxyvitamin D
HES: Hospital episode statistics
PCA: Prescription cost analysis
THIN: The Health Improvement Network
VDD: Vitamin D deficiency
