# Supplementary material for: Costs of vitamin D testing and prescribing among children in primary care
Source: Eur J Pediatr. 2017 Aug 12;176(10):1405–9. doi: 10.1007/s00431-017-2986-9 (PMC5602081; doi:10.1007/s00431-017-2986-9)
Supplement: Supplementary file 1 — (PDF 68 kb) [file 431_2017_2986_MOESM1_ESM.pdf]

Supplementary Table 1 Costs of vitamin D prescriptions and tests in children in primary care, by year between 2000 and 2014.

Year	PY of follow-up	Vitamin D Prescriptions				Vitamin D Tests			Combined cost of vitamin D prescriptions and tests per 100,000 PY, £ (95% CI)
		n	Rate per 100,000 PY (95% CI)	Mean prescription cost, £ (95% CI)[a]	Cost per 100,000 PY, £ (95% CI)	n	Rate per 100,000 PY (95% CI)	Cost per 100,000 PY, £ (95% CI)[b]	
2000	161,897	0	0	-	-	1	0.62 (0.09 to 4.38)	9.27 (1.31 to 65.8)	9.27 (1.31 to 65.8)
2001	180,797	2	1.11 (0.28 to 4.42)	37.7 (12.3 to 63.0)	41.7 (3.41 to 279)	2	1.11 (0.28 to 4.42)	16.6 (4.15 to 66.3)	58.3 (7.56 to 345)
2002	211,593	0	0	-	-	2	0.95 (0.24 to 3.78)	14.2 (3.55 to 56.7)	14.2 (3.55 to 56.7)
2003	222,486	1	0.45 (0.06 to 3.19)	9.10 (9.10 to 9.10)	4.09 (0.58 to 29.0)	7	3.15 (1.50 to 6.60)	47.2 (22.5 to 99.0)	51.3 (23.1 to 128)
2004	235,500	4	1.70 (0.64 to 4.53)	175 (105 to 210)	297 (66.6 to 950)	19	8.07 (5.15 to 12.7)	121 (77.2 to 190)	418 (144 to 1140)
2005	244,548	17	6.95 (4.32 to 11.2)	455 (277 to 653)	3164 (1197 to 7299)	20	8.18 (5.28 to 12.7)	123 (79.1 to 190)	3286 (1276 to 7489)
2006	251,499	18	7.16 (4.51 to 11.4)	188 (156 to 210)	1349 (703 to 2386)	23	9.15 (6.08 to 13.8)	137 (91.2 to 206)	1486 (794 to 2592)
2007	257,336	17	6.61 (4.11 to 10.6)	156 (114 to 196)	1033 (471 to 2085)	44	17.1 (12.7 to 23.0)	256 (191 to 345)	1290 (662 to 2430)
2008	265,314	22	8.29 (5.46 to 12.6)	120 (71.8 to 177)	997 (392 to 2227)	115	43.3 (36.1 to 52.0)	650 (542 to 781)	1647 (934 to 3007)
2009	270,643	65	24.0 (18.8 to 30.6)	201 (136 to 285)	4818 (2563 to 8717)	240	88.7 (78.1 to 101)	1330 (1172 to 1510)	6148 (3735 to 10,226)
2010	278,167	106	38.1 (31.5 to 46.1)	123 (97.4 to 151)	4696 (3067 to 6983)	431	155 (141 to 170)	2324 (2115 to 2554)	7020 (5182 to 9537)
2011	284,678	266	93.4 (82.9 to 105)	75.9 (59.0 to 97.7)	7092 (4887 to 10,298)	733	257 (240 to 277)	3862 (3593 to 4152)	10,954 (8480 to 14,450)
2012	288,818	912	316 (296 to 337)	47.6 (42.1 to 53.5)	15,034 (12,448 to 18,015)	1385	480 (455 to 505)	7193 (6824 to 7582)	22,227 (19,272 to 25,597)
2013	283,990	1604	565 (538 to 593)	34.7 (31.7 to 38.0)	19,617 (17,032 to 22,548)	2064	727 (696 to 759)	10,902 (10,441 to 11,382)	30,519 (27,473 to 33,931)
2014	253,097	1551	613 (583 to 644)	28.4 (26.3 to 30.6)	17,397 (15,347 to 19,700)	1943	768 (734 to 803)	11,515 (11,015 to 12,039)	28,913 (26,361 to 31,739)

CI confidence interval, *PY* person-years.
[a] Confidence intervals around mean prescription costs were calculated using bootstrapping with the percentile method, in view of the skewed nature of the data.
[b] The unit cost of a 25-OH-D test was priced at £15.
