# Supplementary material for: Costs of vitamin D testing and prescribing among children in primary care
Source: Eur J Pediatr. 2017 Aug 12;176(10):1405–9. doi: 10.1007/s00431-017-2986-9 (PMC5602081; doi:10.1007/s00431-017-2986-9)
Supplement: Supplementary file 2 — (PDF 73 kb) [file 431_2017_2986_MOESM2_ESM.pdf]

Supplementary Table 2 Associations between demographic factors and rates of vitamin D testing and prescribing [a]

Characteristic	Vitamin D Testing		Vitamin D Prescribing	
	Adjusted RR (95% CI)	P value [b]	Adjusted RR (95% CI)	P value [b]
Sex		<0.001		<0.001
Male	1		1	
Female	1.49 (1.41-1.57)		1.41 (1.32-1.50)	
Age group		<0.001		<0.001
0-4 years	1		1	
5-9 years	1.62 (1.49-1.77)		2.00 (1.78-2.26)	
10-14 years	2.97 (2.74-3.22)		4.40 (3.95-4.90)	
15-17 years	5.02 (4.62-5.46)		6.90 (6.18-7.71)	
Ethnicity [c]		<0.001		<0.001
White	1		1	
Asian or Asian British	6.22 (5.76-6.72)		9.60 (8.73-10.6)	
Black or black British	3.80 (3.46-4.18)		5.54 (4.93-6.23)	
Mixed	2.55 (2.20-2.94)		2.57 (2.11-3.13)	
Chinese or other ethnic group	3.33 (2.94-3.77)		3.93 (3.37-4.60)	
Area of residence		0.231		0.149
Urban	1		1	
Rural	0.88 (0.71-1.09)		0.85 (0.67-1.06)	
Season [d]		<0.001		<0.001
Summer	1		1	
Autumn	1.00 (0.93-1.08)		0.95 (0.87-1.05)	
Winter	1.00 (0.93-1.08)		1.00 (0.91-1.09)	
Spring	1.17 (1.09-1.26)		1.16 (1.06-1.27)	

CI confidence interval, *RR* rate ratio.

[a] Results of multivariable Poisson regression models of rates of vitamin D testing and prescribing. Models adjusted for all variables listed in the table, and additionally included the general practice as a random effect. Missing data was handled using complete cases analysis. N=526,890.

[b] P values from likelihood ratio tests comparing nested models.

[c] Ethnicity data was taken from the child's THIN or HES record for 82.8% of children. Maternal ethnicity was used as a proxy measure for the remaining 17.2%.

[d] Summer was defined as June to August, autumn as September to November, winter as December to February, and spring as March to May.
